# Dr. Antoine F. Vadeboncoeur

**Published:** 1911-12

**Authors:** 


Dr. Antoine F. Vadeboncoeur of Syracuse, died November 9, 1911, of apoplexy. He was born in Louisville, Quebec, 57 years ago. He was a neurologist and especially interested in the therapeutic value of music.

